# Targeting glioblastoma via intranasal administration of Ff bacteriophages

**DOI:** 10.3389/fmicb.2015.00530

**Published:** 2015-05-27

**Authors:** Eyal Dor-On, Beka Solomon

**Affiliations:** Department of Molecular Microbiology and Biotechnology, George S. Wise Faculty of Life Sciences, Tel-Aviv UniversityTel-Aviv, Israel

**Keywords:** Ff bacteriophages, lipopolysaccharides, glioblastoma, intranasal delivery

## Abstract

Bacteriophages (phages) are ubiquitous viruses that control the growth and diversity of bacteria. Although they have no tropism to mammalian cells, accumulated evidence suggests that phages are not neutral to the mammalian macro-host and can promote immunomodulatory and anti-tumorigenic activities. Here we demonstrate that Ff phages that do not display any proteins or peptides could inhibit the growth of subcutaneous glioblastoma tumors in mice and that this activity is mediated in part by lipopolysaccharide molecules attached to their virion. Using the intranasal route, a non-invasive approach to deliver therapeutics directly to the CNS, we further show that phages rapidly accumulate in the brains of mice and could attenuate progression of orthotopic glioblastoma. Taken together, this study provides new insight into phages non-bacterial activities and demonstrates the feasibility of delivering Ff phages intranasally to treat brain malignancies.

## Introduction

The family of Ff filamentous bacteriophages (phages) consists of three members (f1, M13, and fd) that share 98.5% homology in their DNA and a similar morphology; a flexible filament, about 900 nm long and 6–10 nm in diameter. They infect *Escherichia coli* carrying the F-episome and propagate without causing cell lysis (Rakonjac et al., 2011).

Since the introduction of phage display technique by Smith (1985), Ff phages have been extensively utilized in various biotechnology applications, both *in vitro* and *in vivo*, including in human patients (Pasqualini and Ruoslahti, 1996; Arap et al., 1998; Frenkel and Solomon, 2002; Larocca et al., 2002; Krag et al., 2006; Yacoby et al., 2006; Rakover et al., 2010; Rakonjac et al., 2011; Roehnisch et al., 2014). However, although the interaction between Ff phages and bacteria has been well studied, knowledge of their impact on the mammalian macro-host is rather sparse.

The need to study such potential interactions is underscored by the fact that phages populate different niche in the mammalian macro-host (Kutter, 2005; Letarov and Kulikov, 2009) as well as a growing body of evidence suggesting that some phages, including Ff phages, have the capacity to promote non-bacterial activities, even though they have no tropism to mammalian cells. For example, Ff phages can elicit intense humoral and cellular immune responses and thus, are utilized in vaccination as carriers of foreign motifs as well as adjuvants (Minenkova et al., 1993; Willis et al., 1993; De Berardinis et al., 1999, 2000; Frenkel et al., 2000; Wan et al., 2001; Wu et al., 2002; Prisco and De Berardinis, 2012). In addition, Ff phages have been reported to possess anti-tumorigenic properties; Stimulation of cultured tumor associated macrophages (TAM’s) with lipopolysaccharide (LPS) free, wild-type M13 phages skewed their polarization toward the anti-tumorigenic M1 phenotype and promoted migration of cytotoxic neutrophils in response to factors secreted by stimulated TAM’s (Eriksson et al., 2009). Accordingly, treatment of mice bearing subcutaneous melanoma tumors with tumor specific phages (displaying B16-F10 mouse melanoma specific peptide or HLA-A2 specific Fab) led to an intense anti-tumorigenic response associated with neutrophil infiltration into the tumor microenvironment and prolonged survival (Eriksson et al., 2007).

Intranasal administration is a non-invasive approach which facilitates to bypass the blood–brain barrier and deliver therapeutics directly to the CNS. Drugs administered via the intranasal route avoid hepatic first pass metabolism and have limited effect on periphery organs compared with systematically administered drugs (Thorne et al., 2004; da Fonseca et al., 2011; Henkin, 2011; Lochhead and Thorne, 2012). Surprisingly, Ff phages (MW = 12 × 10^6^ Da) were previously reported to gain access to the CNS of mice when given intranasally. This was demonstrated to depend on their filament structure and was further utilized to deliver anti-β amyloid antibody fragment into brains of APP transgenic mice to facilitate *in vivo* targeting of β amyloid plaques (Frenkel and Solomon, 2002).

Here, we aimed to evaluate the feasibility of treating brain malignancies via intranasal administration of Ff phages using an aggressive murine model of glioblastoma. Glioblastoma and malignant gliomas account for the majority of the malignant primary brain tumors in humans. Current treatment of glioblastoma is based on tumor resection to the extent feasible followed by radiotherapy and temozolomide chemotherapy, yet, tumor recurrence occurs in virtually all cases and the prognosis of glioblastoma patients remains dismal having a median survival of 15 months from day of diagnosis (Omuro and DeAngelis, 2013).

In our attempt to remove bacterial debris from our phage preparations prior to their administration to mice we observed that LPS, a major byproduct of coliphages preparations, could not be completely eliminated. LPS is released to the media upon lysis of Gram-negative bacteria and acts as a powerful activator of innate immune responses (Bryant et al., 2010). In fact, LPS can affect a wide range of biological processes including angiogenesis, tumorigenesis, and metastasis (Mattsby-Baltzer et al., 1994; Harmey et al., 2002; Reisser et al., 2002; Pollet et al., 2003). Picogram concentrations of LPS are sufficient to promote cell activation while high enough concentrations can lead to sepsis and septic shock accompanied by disseminated intravascular coagulation (DIC) and multiple organ failure (Gioannini et al., 2004; Angus and van der Poll, 2013). As such, the removal of LPS from phage preparations has been addressed by several studies (Kutter and Sulakvelidze, 2004; Zakharova et al., 2005; Eriksson et al., 2007; Oslizlo et al., 2011). The conventional technique applied to eliminate LPS from recombinant proteins and phages, follows the phase separation protocol using Triton X-114 (Aida and Pabst, 1990).

In this study, phage purification with Triton X-114 alone or in combination with caesium chloride yielded 1,000–10,000 fold decrease in LPS concentration compared to non-purified (NP) phages [Limulus amebocyte lysate (LAL) assay, results are not shown] but failed to result in LPS-free preparations. We show that Ff phages associate with LPS and that LPS contributes to their anti-tumorigenic activity. Using the intranasal route, we further demonstrate that Ff phages can affect progression of orthotopic glioblastoma.

## Results

### Ff Phages are Carriers of LPS

**FIGURE 1:**
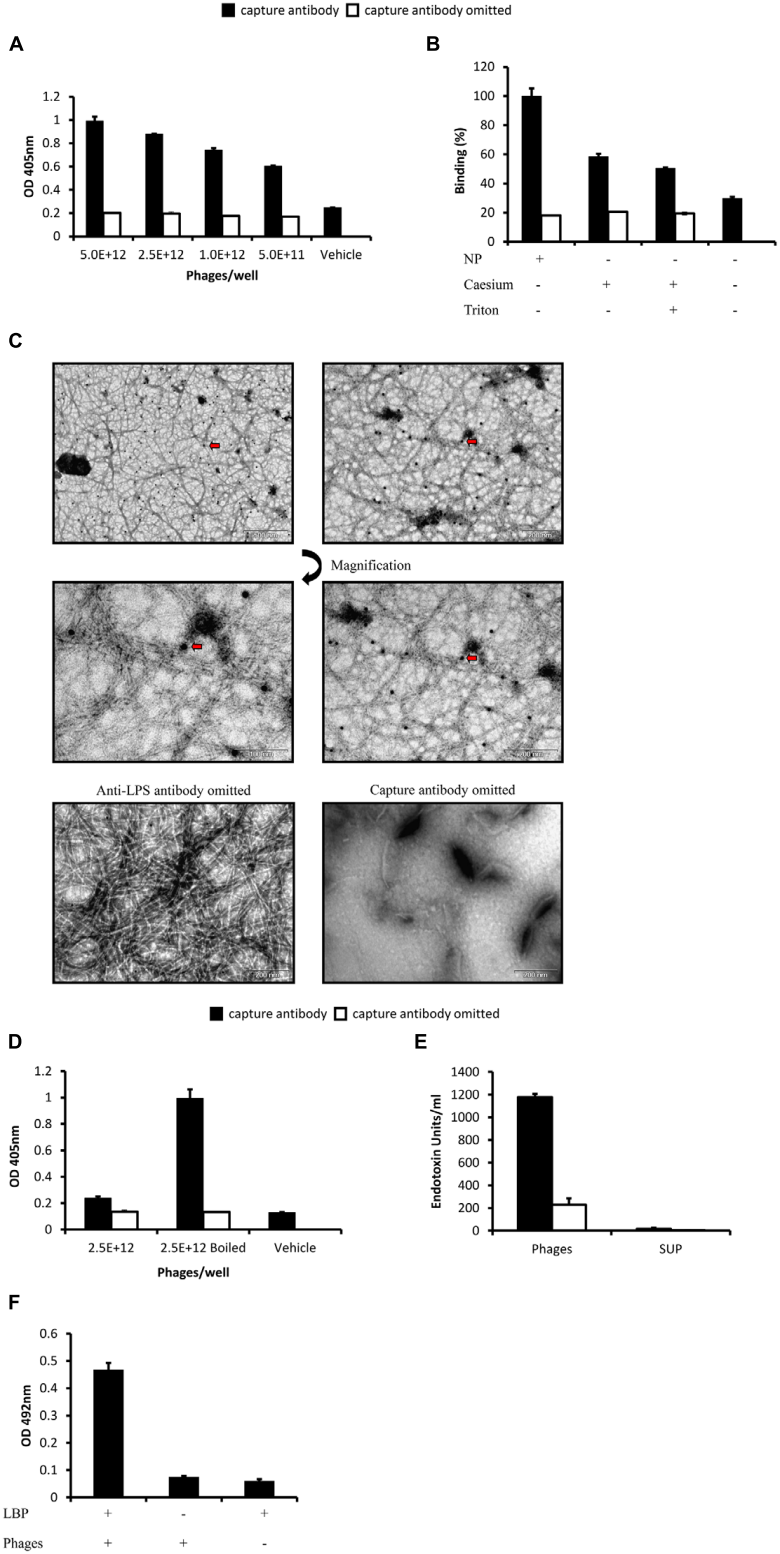
**Detection of lipopolysaccharide (LPS) on the surface of Ff phages. (A)** ELISA plate was coated with anti-p3 antibodies (capture antibody) in coating buffer or coating buffer alone. Different concentrations of non-purified (NP) phages were applied to the plate and LPS was detected by anti-LPS antibodies followed by alkaline phosphatase (AP) conjugated antibodies. **(B)** Sandwich ELISA was performed as in **(A)**, equal concentrations of NP phages (following PEG precipitation), caesium purified phages or caesium, and Triton purified phages were applied to the plate. **(C)** NP phages were immobilized to a nickel grid by anti-p8 capture antibodies, LPS was detected by anti-LPS antibodies followed by gold conjugated antibodies (arrow). Phages were visualized by negative staining. **(D)** Sandwich ELISA was performed as in **(A)**, wells were supplemented with NP phages or equal concentration of NP phages after 3 min incubation at 95°C. The plate was read following short exposure to AP substrate. **(E)** ELISA plate was coated with anti-p3 antibodies, NP phages or bacteria supernatant were applied to the plate and the complex was detached by incubation with NaOH. Endotoxin concentration in the samples was quantified using the limulus amebocyte lysate (LAL) assay. **(F)** ELISA plate was coated with rhLBP then E+13/well NP phages were applied to the plate and were detected by anti-p8 HRP conjugated antibodies. Results are depicted as mean ± SEM.

To investigate whether LPS interacts with Ff phages, NP phages were immobilized to microtiter plates by capture antibodies and exposed to anti-LPS antibodies. LPS was detected on the surface of phages in a dose dependent manner (**Figure 1A**). This was also supported by direct and sandwich ELISA based immunogold transmission electron microscopy (TEM) showing co-localization of LPS with phages (Supplementary Material, **Figure 1C**). Similar to the coat proteins of phages, LPS was mostly detected adjacent to the virion rather than in phage-clear regions (Supplementary Material). As expected, purification of phages with caesium chloride alone or in combination with Triton reduced signal intensity (**Figure 1B**). Yet, even when both techniques were applied, antibodies still detected LPS on immobilized phages. Furthermore, when denatured phages (boiled phages) were used in the same ELISA, detection of LPS was highly increased (**Figure 1D**) plausibly owing to exposure of new phages and LPS epitopes. Interestingly, NP phages or purified phages failed to react with supernatant containing LPS of stationary-phase uninfected bacteria in direct ELISA (data is not shown). Collectively these observations imply that LPS molecules associate with the virion, some in sites that are not surface exposed, yet Ff phages show no affinity to LPS in these experimental conditions.

Whether LPS carried by phages is biologically active depends on its chemical and biophysical structure (Mueller et al., 2004). Using the chromogenic LAL assay we demonstrate that LPS derived from immunopurified phages could exert biological activity (**Figure 1E**). This may also indicate that Ff phages carry LPS aggregates on their surface (Mueller et al., 2004). Furthermore, phages could interact with LPS binding protein (LBP; **Figure 1F**) which binds to LPS aggregates and catalyzes their transfer to CD14 (Hailman et al., 1994; Gioannini et al., 2004; Park and Lee, 2013).

### Ff Phages and Purified LPS Inhibit Subcutaneous Tumor Growth

**FIGURE 2:**
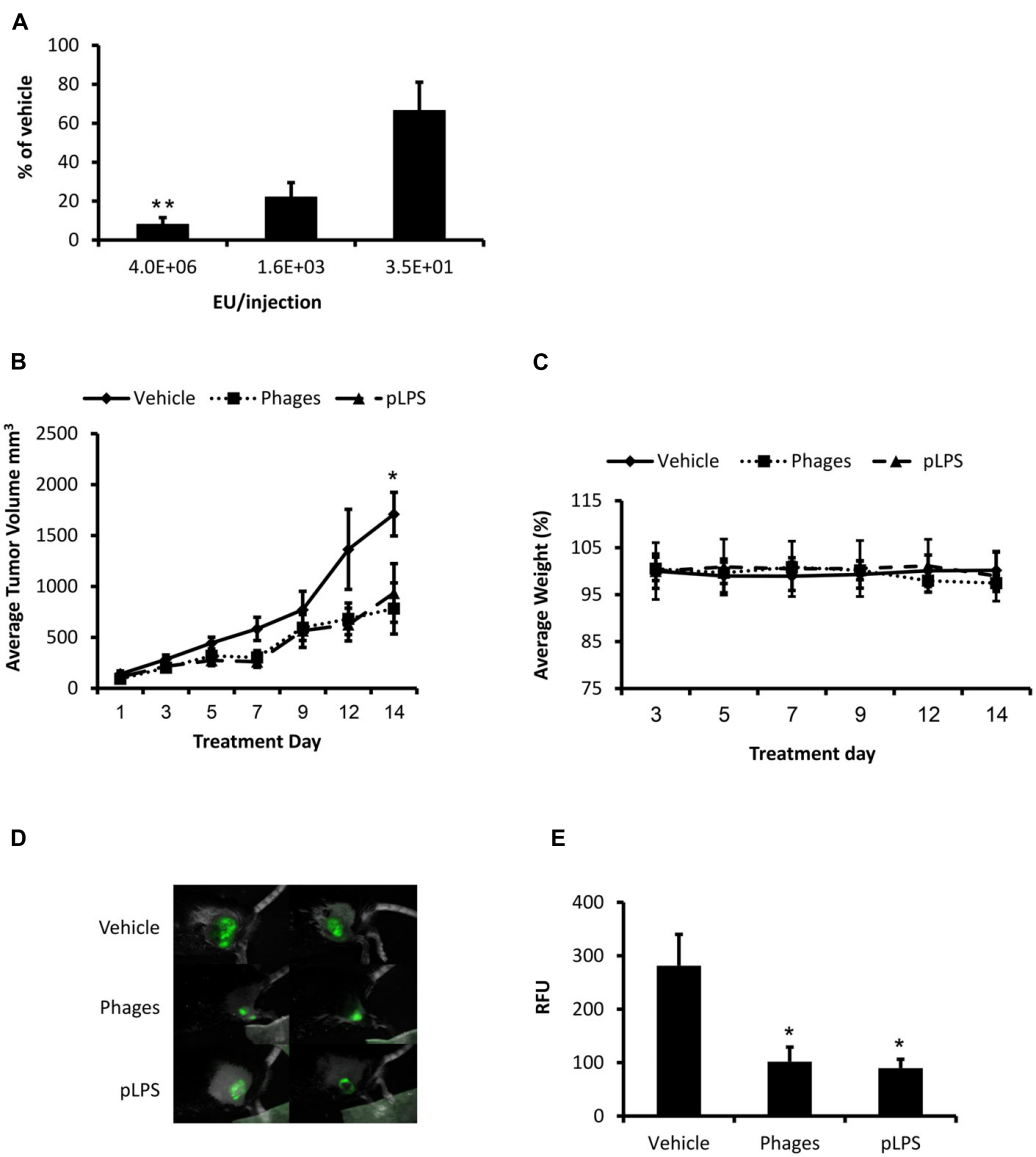
**Ff phages and purified LPS (pLPS) inhibit tumor growth. (A)** C57BL/6 mice (*n* = 5) inoculated subcutaneously with GL261 cells were peritumorally administered with 1.7E+12 phages from preparations containing different endotoxin concentration every second day for approximately 10 days. Tumor dimensions were measured by a caliper and tumor volume was calculated as follows: width2^∗^length/2 (^∗∗^*P* < 0.01 as compared to 3.5E+1 EU/injection, Kruskal–Wallis test followed by Dunn–Bonferroni’s *post hoc* analysis). Results were normalized to the vehicle group. **(B)** C57BL/6 mice (*n* = 5–6) were inoculated subcutaneously with 2E+6 GL261 glioblastoma cells stably expressing green fluorescent protein (GFP). Mice received peritumoral injections every second day of vehicle (PBS), 1.7E+12 Triton purified phages (430 EU/injection), or pLPS (430 EU/injection). Tumor dimensions were measured as in **(A)** (^∗^*P* < 0.05 phages compared to vehicle, unpaired two-tail Student’s *t*-test). **(C)** Change in body weight of mice during the experiment. **(D)** Intravital fluorescence imaging performed 9 days following treatment initiation, presented are representative images. **(E)** GFP intensity (total GFP signal scaled counts/s) is depicted as relative fluorescence units (RFU; ^∗^*P* < 0.05 phages and pLPS compared to vehicle, one-way ANOVA followed by Tukey’s *post hoc* analysis). Results are depicted as mean ± SEM.

We then assessed whether LPS contributes to the activity of phages *in vivo* using an immunocompetent mouse model of glioblastoma (Szatmári et al., 2006). Repeated administration of 1.7E+12 phages from preparations containing different endotoxin concentration to mice bearing subcutaneous GL261 tumors shows that phages could suppress tumor growth, yet the intensity of their anti-tumorigenic activity was positively correlated with the concentration of endotoxin found in the preparation (**Figure 2A**). To better evaluate the involvement of LPS in this activity, mice bearing subcutaneous GL261 stably expressing green fluorescent protein (GFP) were injected peritumorally with phages or purified LPS extracted from naive bacteria (pLPS). Administration of phages or pLPS containing equal endotoxin concentration (430 EU/injection) inhibited tumor growth to a similar extent (54 and 45%, respectively, vs. vehicle at day 14; **Figure 2B**) with no apparent systemic toxicity or reduction in body weight (**Figure 2C**). Consistent with these results, intravital fluorescence imaging performed after 9 days of treatment revealed reduction of 63.7% in total signal from tumors treated with phages compared to administration of vehicle alone (*P* = 0.015; **Figures 2D,E**), which was similar to the effect of treatment with pLPS (68% vs. vehicle *P* = 0.01). This trend was also supported by *ex vivo* fluorescence imaging of tumors at the end of the experiment (data is not shown). Taken together, these findings suggest that the activity of phages in this model was promoted predominantly by the presence of LPS in the preparation.

### Ff Phages Administered Intranasally Accumulate in the Brains of Mice and Inhibit Brain Tumor Progression

**FIGURE 3:**
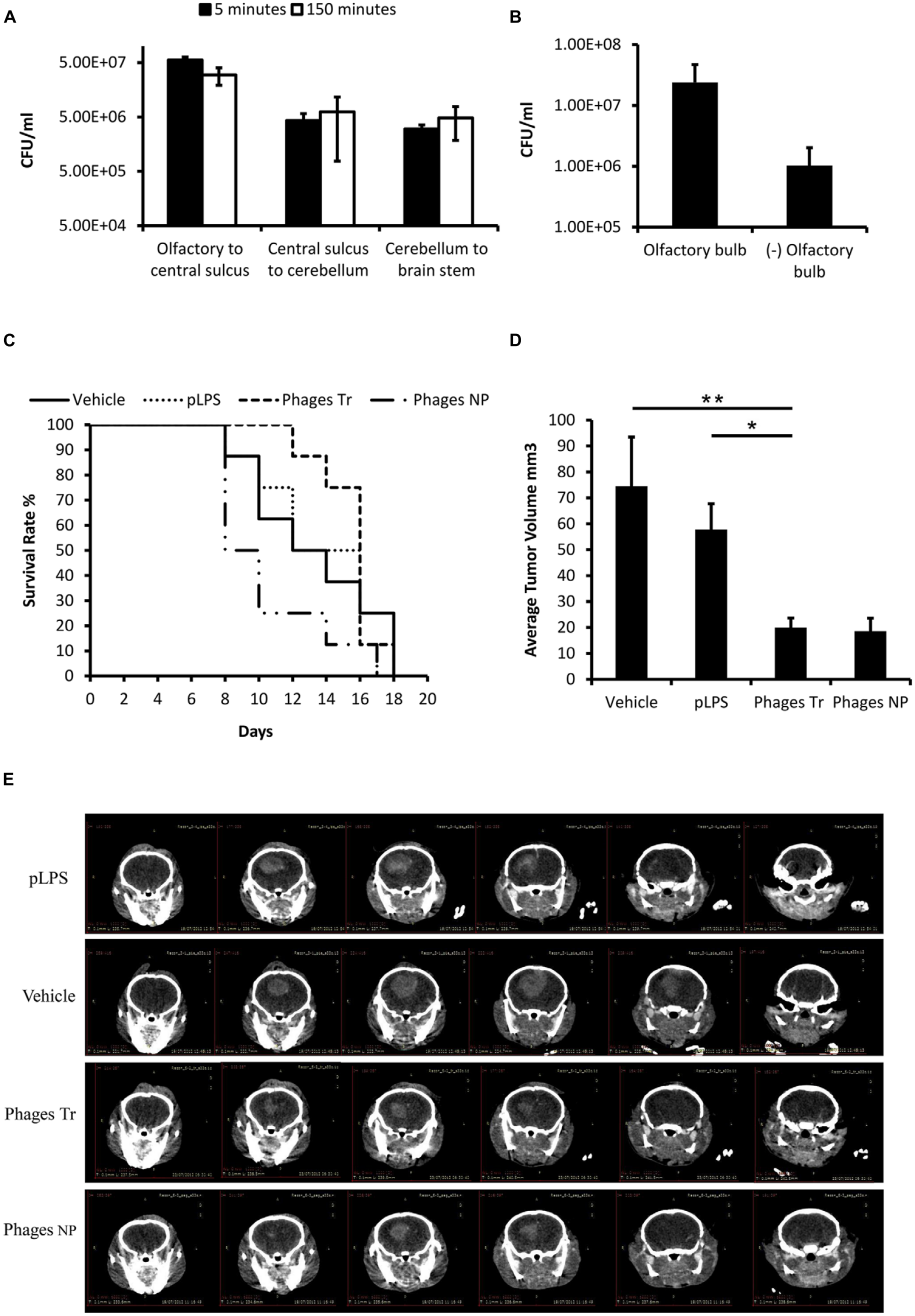
**Ff phages delivered intranasally accumulate in the CNS of mice and suppress progression of orthotopic glioblastoma. (A,B)** Mice were intranasally administered with 2E+12 phages and perfused at the indicated time points. Phage concentration in brain homogenates was evaluated by live counting. Data is presented as colony forming units (CFU) per ml, normalized to tissue weight. **(A)** Phage concentration in different brain regions 5 and 150 min following intranasal administration (*n* = 3). **(B)** Phage concentration in the olfactory bulb and the rest of the brain 1 hour following intranasal administration (*n* = 3). **(C–E)** C57BL/6 male mice bearing orthotopic GL261 GFP tumors were intranasally administered with vehicle alone (PBS), 1.9E+12 Triton purified phages (975 EU/administration), pLPS (975 EU/administration) or 1.9E+12 NP phages (110,500 EU/administration). Treatment started three days following cell inoculation. **(C)** Kaplan–Meier analysis of survival followed by a log-rank test. Decrease of 10% in mice body weight from day of cell inoculation was set as an endpoint, *n* = 8. **(D,E)** CT imaging was performed 2 weeks following cell implantation. Depicted are axonal sections (one representative mouse per group) displayed in 1 mm intervals from frontal (left) to rostral (right). Tumor volume was extrapolated from CT images as described in material and methods. (^∗^*P* < 0.05, ^∗∗^*P* < 0.01, one-way ANOVA was applied on logarithmic transformed data followed by Bonferroni’s *post hoc* analysis), *n* = 5. Results are expressed as untransformed mean values ± SEM.

We previously reported that Ff phages administered via the intranasal route could be detected in sections of the olfactory bulb and hippocampus regions of mice using immunohistochemistry staining (Frenkel and Solomon, 2002). Consistent with these results, here we demonstrate that infective phages could be isolated from both rostral and caudal regions of the brain minutes following intranasal administration (**Figure 3A**), and that phages predominantly accumulate in the olfactory bulb (**Figure 3B**). These observations indicate that phages plausibly utilize the olfactory system to penetrate the brain and that phages remain intact following intranasal delivery.

We then investigated the effect of phages and pLPS given intranasally to mice bearing orthotopic GL261 tumors. Administration of purified phages extended median survival of mice by 33% compared to mice treated with vehicle alone albeit not statistically significant (median survival of mice treated with phages was 16 vs. 12 days of mice treated with vehicle alone *P* = 0.456; **Figure 3C**). Intranasal administration of pLPS containing equal concentration of endotoxin units had virtually no effect on the survival of mice. Computed tomography (CT) scan performed on day 14 shows that mice treated with purified phages had significantly smaller tumors compared to mice treated with vehicle alone (73%, *P* = 0.006) or pLPS (65% *P* = 0.016, **Figures 3D,E**). In contrast, pLPS had only minor, non-significant effect on tumor progression (22%, vs. vehicle, *P* = 1).

Interestingly, treatment of mice with NP phages inhibited intracranial tumors by 75% (**Figures 3D,E**), however, these mice had significantly lower survival rate compared to mice treated with purified phages (*P* = 0.017) and tended to die earlier then mice treated with vehicle alone (**Figure 3C**). These observations suggest that LPS derived from DH12S cells or other factors found in the preparation are toxic in high doses. In support of this, treatment of mice bearing subcutaneous tumors with NP phage preparations produced in DH12S cells was also associated with toxicity and fatalities (data is not shown). This toxicity was diminished following phage purification.

Collectively, these results show that phages exerted an intense anti-tumorigenic activity in the CNS of mice following intranasal administration and mediated superior effect over treatment with pLPS.

## Discussion

In this study, Ff phage processing with Triton X-114 highly reduced LPS contamination but failed to result in LPS free preparations. Similar results were obtained following phage purification with caesium chloride. These observations, supported by TEM and ELISA analysis demonstrating localization of LPS molecules on the surface of NP as well as purified phages, suggest that Ff phages naturally carry LPS on their surface and at least some of these LPS molecules form a stable complex with the virion that cannot be easily dissociated. Of note, LPS was still detected on the virion even when phages immobilized to ELISA plate were repetitively washed with PBS containing tween detergent or when phages were heated to 95°C. Yet, as others have previously reported to obtain LPS free Ff phages (Paschen et al., 2005; Sartorius et al., 2011; Roehnisch et al., 2013), it is possible that optimization of the purification technique used in this study (Mantile et al., 2011; Roehnisch et al., 2014; Branston et al., 2015) could have resulted in further depletion of LPS from our preparations.

Although some coliphages such as T4 and T5 can interact with LPS (Rakhuba et al., 2010), to our knowledge, no such interactions have been reported for Ff phages. Accordingly, we were unable to show phage binding to purified (extracted from the host) or NP LPS (sup containing endotoxin) *in vitro*. As such, Ff phages might complex with LPS non-directly, for example, via outer membrane proteins localized at LPS sites that can link between phages and LPS. Such interactions were previously proposed for the TuII^∗^ and TuIB coliphages with LPS complexes of OmpA and OmpC, respectively (Datta et al., 1977; Yu et al., 1981). Interestingly, TolA which interacts with the N1 domain of p3 during infection has been implicated in the processing of the O antigen and its function is required for surface expression of O-specific LPS and to a lesser extent for the LPS core in *E. coli* (Lubkowski et al., 1999; Gaspar et al., 2000; Vines et al., 2005). However, to our knowledge, direct interaction between TolA and LPS was not reported. Nonetheless, TolA can form complexes with porin trimmers associated to LPS (Derouiche et al., 1996).

The capacity of phages to interact with LBP and the results obtained in the LAL assay further support LPS localization on the surface of phages. These findings also suggest that LPS molecules attached to the virion may participate in immune response *in vivo* and raise the possibility that they might contribute to the immunogenicity attributed to Ff phages. Of note, picomolar concentration of endotoxin (from *Neisseria meningitidis* or *E. coli*) was sufficient to promote secretion of IL-8, a neutrophil chemoattractant, from cultured human embryonic kidney 293 (HEK293) stably transfected with TLR4 (Gioannini et al., 2004; Lin et al., 2004). Therefore, minute amount of LPS localized on the virion can potentially promote immune responses and induce non-bacterial activities in the macro-host. In support of this, here we demonstrate that treatment of subcutaneous GL261-GFP tumors with pLPS or phages containing the same amount of endotoxin suppressed tumor progression to a similar extent. Thus, we propose that in this model the activity of phages was largely driven by LPS. This conclusion is further strengthen by the observation that phage preparations depleted of LPS exhibited diminished anti-tumorigenic activity in the subcutaneous tumor model. Our data extend previous work performed with tumor specific phages (Eriksson et al., 2007, 2009), suggesting that *in vivo*, wild-type Ff phages carrying an effective amount of LPS may also promote significant anti-tumorigenic activity. Indeed, LPS has been reported to induce intense anti-tumorigenic activities in both animal and human studies (Chicoine et al., 2007; Lundin and Checkoway, 2009). Similar to the activities attributed to LPS free Ff phages (Eriksson et al., 2009), LPS was reported to tilt macrophages polarization toward the M1 phenotype (Mantovani et al., 2002; Rey-Giraud et al., 2012) and induce a potent anti-tumorigenic activity associated with neutrophil infiltration into the tumor milieu (Chicoine et al., 2007). Interestingly, both LPS and LPS free Ff phages were demonstrated to mediate their anti-tumorigenic activity, at least in part, via TLR4 (Chicoine et al., 2007; Eriksson et al., 2009) which might explain the similarities in their tumor inhibition mechanism.

Although treatment of mice bearing subcutaneous glioblastoma tumors with phages or pLPS led to similar results, phages suppressed orthotopic glioblastoma significantly better than pLPS. This discrepancy may suggest that following intranasal administration, phages translocate to the brain more efficiently than pLPS and thus, facilitate pLPS accumulation in the brain. In support of this idea, soluble LPS tends to aggregate in aqueous solutions and form micelles and vesicles having diameters in sizes that possibly limit its transport through perineural spaces in the fila olfactoria (10–15 nm; Bergstrand et al., 2006; Mistry et al., 2009). Accordingly, Ff phages that acquired a spheroid morphology (having a diameter of 30–70 nm) were restricted from the brain when delivered intranasally (Frenkel and Solomon, 2002).

As previously described, phages can be genetically modified to display tumor homing motifs and chemically conjugated to cytotoxic drugs (Wu et al., 2002; Eriksson et al., 2007; Bar et al., 2008; McGuire et al., 2014). Such phages when administered non-invasively via the intranasal route, might exhibit superior anti-tumorigenic activities to the wild-type phage. Considering that Ff phages are also easily and inexpensively produced and that phages are natural habitants of the mammalian microflora and thus are relatively safe, utilizing them intranasally might be useful in treating brain malignancies.

## Materials and Methods

### Phage Production

Overnight culture of *E. coli* DH12S (kindly provided by Dr. M. Mevarech, Tel-Aviv University, Israel) transformed with M13KO7 Helper phage (NEB), was diluted 1:100 in fresh 2YT media containing 50 μg/ml Kanamycin and incubated for two nights at 37°C while shaking at 250 RPM. The preparation was centrifuged at 7000 RPM for 20 min and supernatant was supplemented with polyethylene glycol NaCl 1:5 (v/v) to facilitate phage precipitation. Following two nights incubation at 4°C, phages were centrifuged at 9000 RPM for 1 h at 4°C, resuspended in PBS and a second PEG precipitation was performed as described. Phages were filtered through a 0.45 μm filter and titer was measured spectrophotometrically according to the formula: phage particles/ml = (O.D_0_._269nm_-O.D_0_._320nm_)^∗^6^∗^10^16^/vector size (bp).

### Phages Purification with Caesium Chloride

Phages were mixed with 2.4 M caesium chloride solution (in PBS) and ultracentrifuged at 37,000 RPM for 65 h at 4°C to obtain a stable gradient. The fraction containing phages was drawn and caesium traces were eliminated by ultracentrifugation at 50,000 RPM for 4 h at 4°C twice. Phages were resuspended in PBS and filtered.

### Phages Purification with Triton

Non-purified or caesium purified phages supplemented with 1% Triton X-114 in PBS (1 ml) were vortexed for 1 min followed by 5 min incubation in ice. Phages were vortexed again, incubated for 10 min at 56°C and centrifuged at 22,500*g* for 10 min at 37°C. Supernatant was collected and the procedure was repeated (×3). Triton traces were eliminated by gel filtration using a sephacryl S-300 column equilibrated with PBS and connected to an Akta chromatography system. Fractions containing phages were concentrated with a 3 kDa cut-off centricon and filtered. Endotoxin concentrations were measured by the LAL assay (Loanza) according to the manufacturer’s instructions and were in the range of 0.1 to 1 EU per 1E+9 phages following triton purification.

### LPS Extraction

Lipopolysaccharide was purified from DH12S bacteria using the LPS extraction kit (Intron) according to the manufacturer’s instructions with the following modification: washing step with 70% ethanol was performed three times to eliminate impurities.

### Detection of LPS on Phages Surface by Sandwich ELISA

Microtiter plates were coated with anti-p3 antibodies (Exalpha) 1:50 in coating buffer (0.1 M NaHCO_3_, pH 9.6) or coating buffer alone overnight at 4°C. Plates were washed three times with PBST (0.05% Tween) followed by three washes with PBS, blocked overnight and supplemented with phages in PBS 1% milk for 1 h at 37°C (in triplicates). Plates were washed as described and incubated with sheep anti-LPS antibodies (Pierce) 1:200 for 1 h at 37°C followed by incubation with rabbit anti-sheep alkaline phosphatase (AP) conjugated antibodies (Zymax) 1:1000 for 1 h at 37°C. Plates were developed with 4-nitrophenyl phosphate (Sigma) and signal intensity was quantified using an ELISA reader at OD 405 nm.

### Detection of LPS on Phages Surface by Direct and Sandwich ELISA Based Immunogold TEM

Nickel grid was coated with mouse anti-p8 antibodies (GE) 1:50 in PBS overnight at 4°C. The grid was rinsed 5 min in PBS (×3), blocked with 3% skim milk for 1 h at RT and incubated with 2E+13 phages/ml in PBS 1% milk (blocking buffer) for 1 h at RT followed by PBS wash as described.

For detection of LPS, the grid was incubated with sheep anti-LPS antibodies 1:50 followed by incubation with rabbit anti-sheep antibodies 1:50 and then goat anti-rabbit 1:20 gold conjugated (12 nm, Jackson Immunoresearch Laboratories). For direct ELISA immunogold TEM, the grid was coated with 2E+13 phages/ml in PBS overnight at 4°C, then LPS was detected as described. For detection of coat proteins, grids coated with phages were exposed to serum from rabbits immunized with NP phages (our laboratory preparation) followed by incubation with goat anti-rabbit gold conjugated. All antibodies were diluted in blocking buffer and incubation was performed at RT for 1 h. For negative staining the grid was incubated for 30 s with 2% uranyl acetate solution at RT. Analysis was performed using the Jeol JEM 1200EX transmission electron microscope.

### Phages Binding to LBP

Microtiter plates were coated with 4 μg/ml of recombinant human LBP (R&D) in coating buffer or coating buffer alone overnight at 4°C. Plates were washed three times with PBST (0.05% Tween) followed by three washes with PBS, blocked with 3% skim milk and supplemented with phages in PBS 1% milk (w/v) for 1 h at 25°C (in triplicates). Plates were washed as described, incubated with mouse anti-phage (p8) HRP conjugated antibodies (GE) 1:5000 for 1 h at 37°C and developed with o-phenylenediamine (OPD, Sigma). The reaction was terminated with 4N H_2_O_2_ and signal intensity was quantified using an ELISA reader at OD 495 nm.

### Reactivity of Immunopurified Phages in the LAL Assay

Microtiter plates were coated with anti-p8 antibodies (GE) 1:50 in coating buffer or coating buffer alone overnight at 4°C. The plate was washed three times with PBST (0.05% Tween) followed by three washes with PBS, blocked with 3% skim milk overnight and supplemented with phages (2E+12/well) or bacteria supernatant (from an overnight culture of naïve cells, filtered through a 0.45 μm filter) in blocking buffer. The plate was incubated for 1 h at 37°C and rinsed thoroughly as described. To detach immobilized phages, the plate was incubated with 50 mM NaOH for 24 h. Samples containing NaOH were serially diluted in endotoxin free water (Biological Industries) and tested in the LAL assay.

### Cell Culture

GL261 cell line (kindly provided by Dr. G. Safrany department of molecular and tumor radiobiology, Frederic Joliot-Curie Institute, Hungary) was grown in Dulbecco’s modified Eagle’s medium (DMEM, Biological Industries) containing 10% fetal calf serum, 0.3 mg/ml L-glutamine, 100 units/ml penicillin and 0.1 mg/ml streptomycin. GL261-GFP cells were cultured in the same medium supplemented with 0.5 mg/ml hygromycin (Sigma). Cells were grown at 37°C in 5% CO_2._

### Construction of GL261-GFP Stable Line

Supernatant of 293T cells containing MLV viruses carrying the GFP gene was kindly provided to us by Dr. E. Bachrach (department of cells research and immunology, Tel-Aviv University, Israel). The supernatant was diluted 1:2 in DMEM medium supplemented with Polybrene at a final concentration of 8 μg/ml. The medium was added to GL261 cells at 60% confluence in 24-well plates and infection was carried out for 2 h at 37°C. The medium was replaced with fresh growing medium and following 48–72 h the culture was supplemented with 1 mg/ml hygromycin. Three days later, cells were diluted and reseeded to obtain single cell colonies. GFP positive colonies were isolated and a single clone was chosen for the rest of the work.

### GL261 Tumor Model

All animal studies were approved by the Institutional Animal Care and Use Committee (approval number: L-10-029).

C57BL/6 (3 months old) female mice were subcutaneously inoculated in their flank with 2E+6 GL261 or GL261-GFP glioblastoma cells suspended in PBS. When tumors were palpable mice were divided into treatment groups with an average tumor volume of 100 mm^3^. Mice received peritumoral injections of phages, pLPS or vehicle alone in a total volume of 0.1 ml every second day. Tumor dimensions were measured by a caliper and tumor volume was calculated as follows: width2*length/2. For measurement of fluorescence signal, mice were anesthetized by intraperitoneal injection of Ketamine/Xylazine (100 mg/kg and 20 mg/kg body weight, respectively), treated with a depilatory cream (Veet) and imaged using the Maestro *in vivo* Imaging System (CRi, Inc.). A band-pass filter from 445 to 490 nm and a long-pass filter over 515 nm were used for emission and excitation light, respectively. The tunable filter was automatically stepped in 10-nm increments from 500 to 800 nm whereas the camera captured images at each wavelength interval with constant exposure. Skin autofluorescence and undesired background signals were eliminated by spectral analysis and linear unmixing algorithm. Mice were weighted every treatment day.

### Biodistribution of Phages in Mice Following Intranasal Administration

C57BL/6 mice were intranasally administered with NP 1E+12 phages suspended in 10 μl PBS through each nostril. Immediately before the indicated time points, mice were overdosed with intraperitoneal injection of Ketamine/Xylazine and perfused through the heart with saline. Organs were excised (surgical tools were cleaned with soap, distilled water and ethanol between dissections), supplemented with ice cold PBS (1 ml per 5 g) containing protease inhibitors (Roche) and homogenized using a mechanical homogenizer. Phages concentration was evaluated by live counting as follows: *E. coli* TG1 cells at late log were incubated with homogenate samples for 1 h at 37°C. The inoculum was serially diluted and samples were seeded on petri dishes containing 50 μg/ml Kanamycin. Following overnight incubation, number of colonies was manually counted.

### Orthotopic Glioblastoma Model

Mice were anesthetized with intraperitoneal injection of Ketamine/Xylazine and placed in a Kopf Stereotaxic Alignment System. An approximately 1 cm-long cut was made in the scalp, to expose the skull and a total of 10^5^ GL261-GFP cells in 3 μL PBS were injected 1 mm posterior and 1.5 mm lateral from the bregma at a 3 mm depth from the skull surface. Cells were injected using a Hamilton syringe at a rate of 1 μl/min. In order to avoid backflow, the needle was left for an additional 1 min before being gradually removed. The scalp tissue was glued and the mice were allowed to recover in their cages. Intranasal treatment was given every second day without anesthesia, in a total volume of 10 μl in each nostril, starting 3 days following cell injection. Mice that lost 15% of their initial body weight or demonstrated severe clinical symptoms (epileptic seizures or inability to move) were sacrificed by CO_2_ inhalation.

### Survival Analysis

Following intracranial inoculation, mice were monitored and weighted daily. Survival endpoint was set to 10% loss of body weight from day of cell inoculation or when mice demonstrated severe clinical symptoms as described.

### Computed Tomography (CT) Scan and Brain Tumor Volume Analysis

Mice were anesthetized with an intraperitoneal injection of Ketamine/Xylazine and CT images were acquired with a high-resolution, low-dose x-ray scanner by a skilled technician. Tumor volume was calculated as follows: in a particular section x, the minor axis and the major axis of the tumor were measured using the Radiant DICOM software and the area (AE) was calculated as follows: AEx = π(major axisx) (minor axisx)/4. Next, tumor volume was calculated using the formula: V = I(AE1 + AE2 +… AEn), where I = section increment (0.8 mm) and n = the number of sections containing tumor (length of *z* axis = I n). Additional details of the volume calculation method have been previously published (Winer-Muram et al., 2002).

### Statistics

The SPSS statistics software (version 21) was used for statistical analysis. Normality distribution and homogeneity of variances were assessed by the ShapiroWilk’s test and Levene’s test, respectively. Significance was evaluated by an unpaired, two-tail Student’s *t*-test. For multiple comparisons, one-way analysis of variance (ANOVA) was performed followed by Tukey’s or Dunn–Bonferroni’s *post hoc* analysis. Alternatively, the non-parametric, Kruskal–Wallis test was performed, followed by Dunn–Bonferroni’s *post hoc* analysis. Survival experiments were analyzed by the Kaplan–Meier’s method followed by a log-rank test. Results were considered significance at *P* < 0.05.
