# Supplementary figures and images for: Targeting glioblastoma via intranasal administration of Ff bacteriophages
Source: Front Microbiol. 2015 May 27;6:530. doi: 10.3389/fmicb.2015.00530 (PMC4445050; doi:10.3389/fmicb.2015.00530)

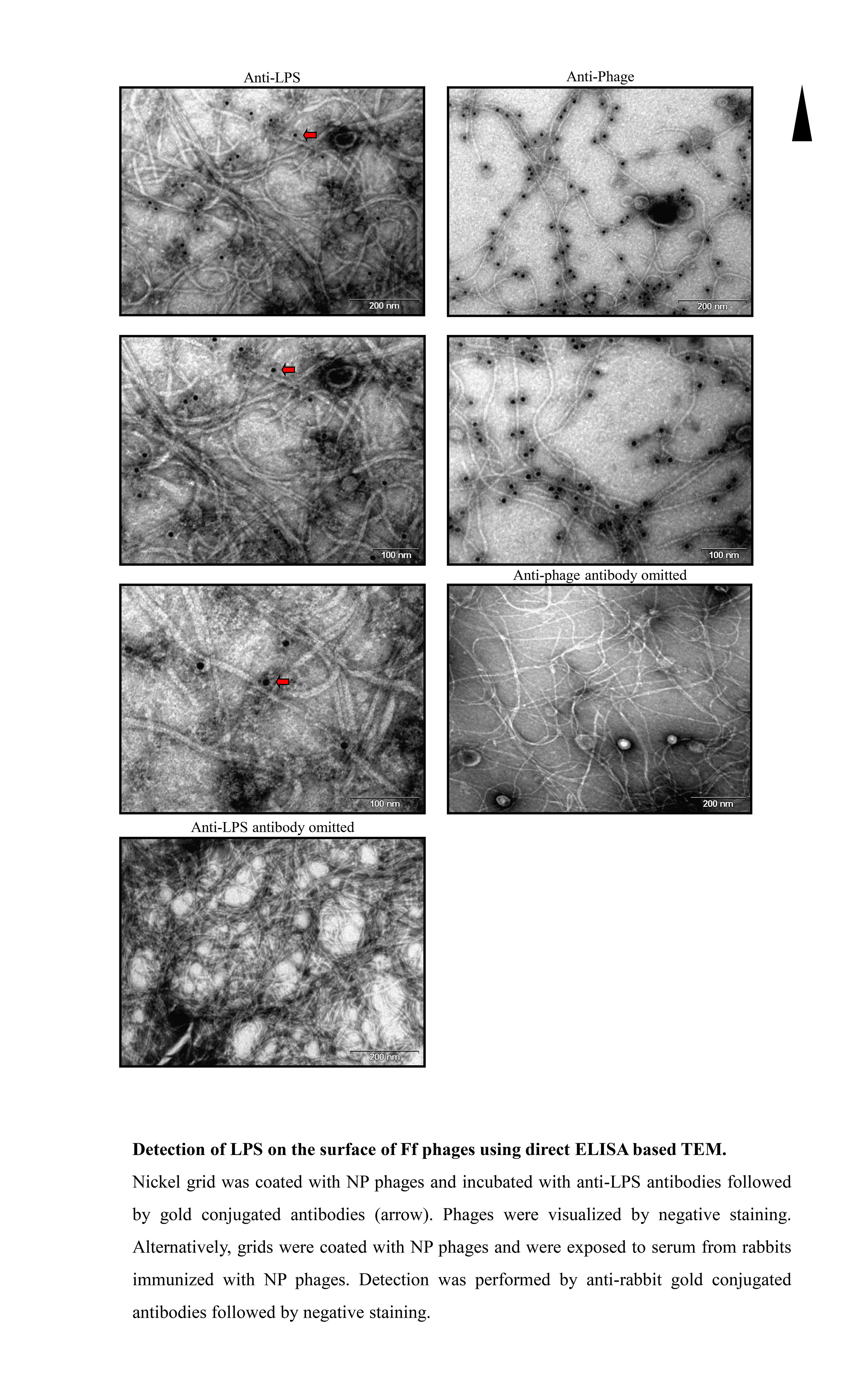

Supplement: Supplementary file 1 [file Image_1.JPEG]
